# Association of diabetes mellitus and structural changes in the central nervous system in children and adolescents: a systematic review

**DOI:** 10.1186/s40200-017-0292-8

**Published:** 2017-03-03

**Authors:** Ata pourabbasi, Mehdi Tehrani-Doost, Soqra Ebrahimi Qavam, Seyed Masoud Arzaghi, Bagher Larijani

**Affiliations:** 1 Diabetes Research Center, Endocrinology and Metabolism Clinical Sciences Institute, Tehran University of Medical Sciences, Tehran, Iran; 2 Department of Psychiatry, Rouzbeh Hospital, Tehran University of Medical Sciences, Tehran, Iran; 3 Faculty of psychology and education, Allameh Tabataba’ee university, Tehran, Iran; 4 Elderly Health Research Center, Endocrinology and Metabolism Population Sciences Institute, Tehran University of Medical Sciences, Tehran, Iran; 5 Endocrinology & Metabolism Research Center, Endocrinology and Metabolism Clinical Sciences Institute, Tehran University of Medical Sciences, Shari’ati Hospital, North Kargar St., Tehran, Iran

## Abstract

**Background:**

The relationship between diabetes and academic performance have been of great interest to researchers during the year to date. Many studies have been conducted to discover this relationship during three recent decades. But, evaluation of the structural changes of brain in the context of diabetes is of paramount importance especially in children and adolescents.

**Methods:**

This study is a systematic review conducted to investigate the structural changes in the central nervous system in children and adolescents living with diabetes. Among about 500 papers published in this area in Pubmed and SCOPUS, 13 articles in the field of assessing structural changes in the central nervous system in children and adolescents with diabetes mellitus were entered into the evaluation process.

**Results:**

As can be seen in these studies, a huge proportion of structures of the central nervous system have been affected by diabetes that include different areas of gray and white matters. In the majority of these studies, it has become clear that high glycemic changes, especially recurrent hyperglycemic attacks are very seriously associated with structural changes in the brain.

**Conclusion:**

It seems the findings of this review can positively aid other researchers to develop medical guidelines to prevent or resolve the brain changes in central nervous structure and consequently cognitive impairments in children and adolescents.

## Background

The relationship between diabetes and academic performance have been of great interest to researchers during the year to date. Many studies have been conducted to discover this relationship during three recent decades [1]. Also, other cognitive functions have been explored besides this relationship in the coming years including memory, attention and executive functions [2] which are some controversies in their relation with diabetes. However, very few studies have addresses the relationship between diabetes and cognitive impairment, academic performance, and its compliance with the structure of the nervous system [3]. In addition, the majority of these few studies are mostly conducted on older adults so that children and adolescents are thereby neglected.

However, with the development of functional imaging techniques in recent years, the changes in the performance of different areas of the central nervous system in children and adolescents with diabetes are evaluated with a greater precision [4]. But, evaluation of the structural changes of brain in the context of diabetes is of paramount importance. The detection of such relationship may offer an open window to identify the mechanism of cognitive changes in children and adolescents with diabetes and to design effective interventions to prevent these changes. The authors of the present paper are trying to prevent structural changes in children and adolescents living with diabetes using this pathway.

Pediatric neuroimaging is faced by major challenges, among which one can name having no tendency of children for different imaging devices due to some fears [5]. On the other hand, despite various studies in this field, different ethical considerations come into play when implementing brain imaging in children and adolescents with diabetes as well as in healthy individuals [6].

Therefore, aiming at evaluating the relationship between diabetes and structural changes in the central nervous system in children and adolescents, the authors of this paper decided to conduct a systematic review to provide a response to the research question. By this systematic review and also considering executive and ethical issues, they hope there is no need to design and run further researches in order to evaluate the relation between diabetes and brain structural changes in children and adolescents.

For certain, finding the answers to these questions may be an effective step in alleviating the diabetes complications on the brain structure and subsequent cognitive functions in children and adolescents with diabetes.

## Methods

This study is a systematic review conducted to investigate the structural changes in the central nervous system in children and adolescents living with diabetes.

### Search strategy

In order to search the published research until the beginning of November 2016, the authors used all articles published in national and international journals indexed in the scientific databases of Pubmed and SCOPUS, as two large and prestigious sources of biomedical and life sciences journal literature. The articles were found using key words or their combinations in English including, neuroimaging, brain change, brain structural, brain map, and diabetes.

### Study selection

The inclusion criteria used to select articles were publication in the last 10 years, publication in English, research on humans, sample aged between 0 and 18 years (in PubMed) and Category of children and adolescents (in SCOPUS).

A list was prepared by the researcher including the relevant titles and abstracts of all papers available in the above mentioned databases, and each were independently studied in order to select the relevant topics. Afterward, the repeated studies were eliminated followed by checking for titles, author(s) names, publication year, Number, and name of journal. Also, based on the titles of the articles, irrelevant unrelated articles to the subject of the present study were excluded from further assessment.

### Quality Assessment

Followed by determining the relevant studies in terms of titles and abstracts, the researchers used STROBE checklist (i.e. strengthening the reporting of observational studies in epidemiology) which is a standard checklist to evaluate the selected papers. Articles given at least score 40 points according to the checklist questions were entered into the research, and their associated data were extracted for the systematic review.

### Data Extraction

In order to extract data from the selected studies, the researchers evaluated them in terms of some details, including titles, first author’s name, year of study, type of study, age group, sample size, type of imaging tool used, and the structural changes observed in the imaging

## Results

### Search process

**Fig. 1:**
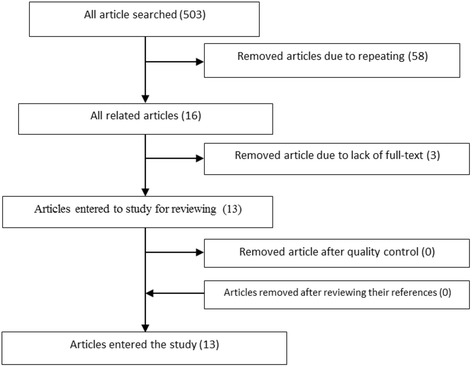
Flowchart of search strategy and the evaluation of papers

Using relevant keywords and making a search with high sensitivity, 503 papers published until the beginning of November 2016 were identified. Of these articles, 58 articles were deleted due to duplication from the former evaluated banks. After the removal of titles unrelated to the subject of the study, 16 related articles remained. It should be noted that a large number of published articles on the subject of diabetes insipitus were excluded at this stage. For this reason, a considerable number of articles were excluded at this stage. Also, those articles examining the structural changes in the brain due to diabetic ketoacidosis, as one of the most important neurological complications in pediatric diabetes, were also excluded. Three other papers were eliminated due to lack of access to the full-text of article despite sending email to the correspondence author. Finally, 13 articles in the field of assessing structural changes in the central nervous system in children and adolescents with diabetes were entered into the evaluation process. The flowchart of search strategy and the evaluation of papers are presented in Fig. 1.

A summary of the key findings are as follows (Table 1):

**Table 1:** Main characteristics and key findings of reviewed studies

No.	Author	Title	Year	Study type	Samples	ASSESSMENT tool	Main findings	Authors’ comments
1	Rofey, D. L.	Brain volume and white matter in youth with type 2 diabetes compared to obese and normal weight, non-diabetic peers: A pilot study	2015	Case–control	15 male adolescent aged 12–21 years	MRI	Higher volume of caudate core in the group with normal weight compared to the obese and diabetic groups. Higher Thalamic volume in both normal and obese groups. Evaluating white matter, diabetics had the highest deficit in Functional anisotropy Volume (FA). But such differences were not significant.	Brain morphology can be different between youth with T2DM, obese youth, and normal-weight controls.
2	Mauras, N.	Longitudinal assessment of neuroanatomical and cognitive differences in young children with type 1 diabetes: association with hyperglycemia	2015	cohort	214 Children aged 4–10 years, 142 diabetic and 72 healthy children	Structural MRI at baseline and 18 months later with cognitive assessment	A slower rate of growth in the volume of white matter (in splenium of corpus callosum, bilateral superior-pareital lobe, bilateral avterior forceps and inferior-frontal fasciculus) and gray matter (in left precuneus, right temporal, frontal and parietal lobes and right medial-frontal cortex) in diabetic group. These changes are associated with cumulative hyperglycemia and large changes in glucose levels; But, was not associated with hypoglycemia. No significant differences in the growth of white and gray matters involved in sensorimotor and cognition processes after 18 months in diabetic children	chronic hyperglycemia can have negative effects on the developing brain
3	Marzelli, M. J.	Neuroanatomical correlates of dysglycemia in young children with type 1 diabetes	2014	Case–control	142 diabetic and 68 healthy children with the average age of 7 Yrs(1.7)	Structural MRI	Reduced gray matter volume in the bilateral occipital and cerebellar regions Increased volume of gray matter in the left inferior prefrontal, insula, and 5temporal pole The volume of gray matter in some areas is inversely correlated with HbA1C levels. Similarly, the volume of gray matter in certain areas of bilateral medialis inversely associated with the duration of diabetes. The volume of white matter in the left anterior frontal lobe has a negative correlation with the duration of diabetes	Early-onset T1D affects regions of the brain involving in typical cognitive development.
4	Barnea-Goraly, N.	Alterations in white matter structure in young children with type 1 diabetes	2014	Case–control	127 diabetic and 67 healthy children aged 4–10 year	MRI	Significantly reduced axial diffusivity (AD) in widespread brain regions recurrent hyperglycemia is significantly correlated with structural changes in white matter an inverse correlation Duration of diabetes with the size of AD and RD and a positive correlation with FA values in widespread brain regions Correlation between earlier age of diabetes onset and lower FA and higher RD values	vulnerability of the developing brain in diabetic children were in correlation with chronic hyperglycemia and glucose variability
5	Antenor-Dorsey, J. A.	White matter microstructural integrity in youth with type 1 diabetes	2013	Case–control	72 diabetic with the average age of 16.8 years (2.9) and 30 healthy controls with the average age of 15.9 years (3.4)	Diffusion tensor imaging using 2 complimentory approaches: region of interest and voxelwise tract-based spatial statistics	Lower fractional anisotropy in the superior parietal lobule Reduced mean diffusivity in the thalamus Correlation between history of three or more attacks of severe hyperglycemia and reduced anisotropy, increased diffusivity in the superior parietal lobule, and increased diffusivity in the hippocampus	Superior parietal lobule, hippocampus, and thalamus are sensitive to glycemic extremes during brain development.
6	Aye, T.	White matter structural differences in young children with type 1 diabetes: a diffusion tensor imaging study	2012	Case–control	22 diabetic children Aged 3–10 year and 14 healthy subjects	MRI Diffusion tensor imaging	Lower values of axial diffusivity in temporal and parietal lobe regions: internal capsule, body of the corpus callosum, right singulate gyrus and posterior parietal lobe in diabetics Statistically significant difference in fractional anisotropy and Radial diffusivity Straight and significant correlation between HbA1C and RD in some areas of the white matter A negative significant correlation between the level of HbA1C and FA in right internal capsule, anterior forceps, inferior fronto-occipital fasciculi, and splenium of CC	Diabetic children had significantly different white matter structure compared with controls which correlated with HbA1c values.
7	Perantie, D. C.	Prospectively determined impact of type 1 diabetes on brain volume during development	2011	cohort	75 diabetic children with the average age of 12.5 years and 25 healthy controls	MRI in two time periods within 2 years	No significant differences between the two groups in terms of variations in the overall brain volume and voxel-wised was seen in two years. A higher reduction of the overall volume of gray matter in the diabetic group with more hyperglycemic attacks. A higher reduction of the overall volume of gray matter in occipital and parietal areas of individuals with history of recurrent severe hypoglycemia	hyperglycemia and severe hypoglycemia may lead to subtle deviation from normal developmental trajectories of the brain in diabetic children.
8	Aye, T.	The feasibility of detecting neuropsychologic and neuroanatomic effects of type 1 diabetes in young children	2011	Case–control	28 diabetic children aged 3–10 year and 17 healthy controls	MRI	The volume of white matter did not have the expected volume growth rate to fit age in diabetic patients compared to healthy subjects. Similar trend was observed in hippocampal volume; however not seen in the gray matter and the amygdala Reduced volume of gray and white matters in children with diabetes with a history of seizures, with no changes in hippocampal volume. HbA1C levels did not show any correlation with changes in the volume of brain structures.	Early signs of neuroanatomical variation may be present in diabetic children.
9	Yau, P. L.	Preliminary evidence for brain complications in obese adolescents with type 2 diabetes mellitus	2010	Case–control	18 adolescents with type 2 diabetes and 18 healthy controls	MRI	There was no difference in the volume of gray matter. But, smaller white matter and larger CSF space in the brain and frontal lobe were seen in people with diabetes compared to healthy subjects	Brain abnormalities may result from a combination of minor vascular changes, glucose and lipid metabolism abnormalities.
10	Hershey, T.	Hippocampal volumes in youth with type 1 diabetes	2010	Case–control	95 diabetic and 49 healthy children and adolescents aged 7–17 years	MRI	There was no significant difference in the volume of the whole brain and hippocampal volume between diabetes and healthy groups. The correlation between the number of severe hypoglycemic attacks and the growth of hippocampal gray matter volume, especially in the group with three or more attacks	Hippocampus Enlargement may be due to a pathological reaction to hypoglycemia during brain development.
11	Northam, E. A.	Central nervous system function in youth with type 1 diabetes 12 years after disease onset	2009	Historical cohort	106106 diabetic adolescents aged 20.5 years with a history of diagnosed diabetes 12 years ago (4.3) and 75 health controls aged 21 year (3.8)	MRI	Reduced volume of gray and white matters in different areas of Hypoglycemia is a predictor of reduced gray matter volume in the thalamus. Also, poorly controlled metabolic has been shown correlated with the reduced T2 in Thalamus.	Several neuropathological processes including gliosis, demyelination, and altered osmolarity maybe occur during diabetes in children
12	Ho, M. S.	Prevalence of structural central nervous system abnormalities in early-onset type 1 diabetes mellitus	2008	Cross sectional	Diabetic children with a history of diagnosed diabetes before 6 years of age. 32 cases with a history of severe hypoglycemia and seizures and 30 children without a history of seizures	MRI	Structural abnormalities of the central nervous system in 29% of samples such as Chiari malformation, cyst, developmental venus anomalies, and incidental benign tumor Lower density of gray matter in samples with a history of seizures No differences in hippocampal volume were observed between the groups with and without seizures.	Early-onset severe hypoglycemia can affect on gray matter volume.
13	Perantie, D. C.	Regional brain volume differences associated with hyperglycemia and severe hypoglycemia in youth with type 1 diabetes	2007	Case–control	108 diabetic and 51 healthy children and adolescents aged 7–17 years	MRI	No significant difference in the white and gray matters volumes was observed between diabetic and healthy groups. Lower gray matter volume in some areas in patients with diabetes with a history of hypoglycemia Association of recurrent hyperglycemia with Lower gray matter volume in the right cuneus and precuneus, higher volume of gray matter in the right frontal middle gyrus, and lower volume of white matter in the right superior parietal WM The early onset of diabetes is associated with a larger volume of white matter in the left precuneus region.	some different relationships were found between hypo- and hyperglycemia and regional brain volumes in diabetic youth.

### Evaluation of central nervous structures in patients with diabetes compared to healthy subjects

In the majority of conducted studies so far, a group of children and adolescents with diabetes have been compared with a healthy peer where in the majority of them several differences in neural structures have been reported in patients with diabetes compared to the healthy individuals. In the study of Rofey et al. conducted on 15 male adolescents aged 12–21 years in three groups including, diagnosed with type 2 diabetes, obese non-diabetic, and normal- weight nondiabetic, it was found that the volume of caudate in the group with normal weight was significantly higher compared to the obese and diabetic groups. Thalamic volumes in both normal and obese were higher in comparison with diabetics. Although not statistically significant, but such differences were found in some other regions, including the hippocampus, nucleus accumbens, amygdala, putamen, and pallidum. In evaluating white matter, diabetic group were reported to have the highest deficit in terms of functional anisotropy volume (FA). However, such differences were not significant [7].

In another study on 142 children with diabetes aged 4 to 10 years and 72 healthy subjects, Mauras et al. evaluated the structure of the central nervous system using MRI during two separate phases, at baseline and after 18 months. They drew the conclusion that children with diabetes showed a slower growth rate in parts of the gray and white matters volume compared to healthy subjects. Gray matter areas included left precuneus, right temporal, frontal and parietal lobes and right medial-frontal cortex, and white matter areas included splenium of corpus callosum, bilateral superior-pareital lobe, bilateral avterior forceps, and inferior-frontal fasciculus. After 18 months, diabetic children revealed significant differences compared to their healthy peers in the development of white and graymatters involved in the process of sensorimotor and cognition [8].

In another study on 142 children with diabetes and 68 healthy controls with an average age of 7 years (1.7), Marzelli et al. reported the reduced volume of gray matter in the bilateral occipital and cerebellar regions and increased volume of gray matter in left inferior prefrontal, insula, and temporal pole in children with diabetes compared to their health peers [9]. Also, in another study to assess the changes in white matter of brain in diabetic children, Barnea-Goraly et al. reported significantly reduced axial diffusivity (AD) in widespread brain regions [10].

In a similar study conducted by Antenor-Dorsey et al. to evaluate the microstructural changes of white matter in children with diabetes, lower fractional anisotropy in the superior parietal lobule and reduced mean diffusivity in the thalamus in the diabetic group were observed compared to healthy subjects [1]. Also, Aye et al. in their study on 22 diabetic children aged 3–10 years and 14 healthy subjects reported lower doses of axial diffusivity in temporal and parietal lobe regions: internal capsule, body of the corpus callosum, right singulategyrus and posterior parietal lobe in diabetic group than the controls. They also revealed significant differences in the fractional anisotropy and radial diffusivity in diabetic and healthy groups [11].

In another study, they also explored the structural differences in the brains of children with diabetes and evaluating 28 children with diabetes reported that after controlling for age and sex. The volume of white matter in diabetics didn’t show the expected growth rate according to age compared with controls. The same trend was observed in hippocampal volume. But nothing was reported in the gray matter and the amygdala [12].

Developing a cohort study, Perantie et al. compared 75 diabetic children and adolescents with an average age of 12.5 years with 25 healthy subjects in terms of structural central nervous system at two time intervals in the framework of a two year study. Finally, they reported no significant differences in the overall brain volume and voxel-wised changes between the groups in two years [13]. In another cross-sectional study on the diabetic children without evaluating changes over time, no significant difference between diabetic and healthy groups in the white and gray matters volume is reported [14].

In a different study, Yau et al. conducted a different study to analysis the brain structure of 18 obese adolescents with type 2 diabetes and 18 healthy obese adolescents in gray matter volume where no difference in the gray matter volume was observed. However, a smaller white matter, and larger CSF space was observed in the whole and in frontal lobe of people with diabetes compared to healthy subjects [15].

In addition, Hershey et al. in their study of diabetic children aged 7–17 years showed that after matching for the age and sex no significant difference in the whole brain and the hippocampus of diabetic and healthy children were observed [16]. The reduced volume of gray matter in bilateral thalami, right parahippocampal gyrus, right insular cortex and reduction of white matter volume in bilateral mesial temporal lobes (parahippocampal region), left temporal lobe, left middle frontal area in children and adolescents with diabetes compared to healthy subjects is demonstrated in the study of Northam et al.

This study was conducted on 106 diabetic patients aged 20.5 on the average diagnosed with diabetes since 12 years ago and 75 healthy subjects aged 21 (3.8) on the average. And in this respect, this research has important differences with other studies in which the long-term complications of diabetes on brain structures can be well demonstrated [17]. But, Ho et al. conducted a study on diabetic children diagnosed with diabetes before 6 years age aiming to assess the structural abnormalities in the central nervous system of children with diabetes. In this study, abnormalities in the central nervous system was observed in 29% of samples which included Chiari malformation, cyst, developmental venus anomaies, and incidental benign tumor. However, the prevalence of such abnormalities was not well compared with the health subjects group [18].

### Relationship between structural changes in the central nervous system and indicators of glycemic control

In the majority of studies been made to evaluate the relationship between diabetes and the structural changes in the central nervous system its attempted to study the structural changes in the brain and the status of glycemic control in diabetic patient in addition to comparing diabetic children with healthy subjects. These studies, has put forward a clear illustration on the important facts regarding the role of glycemic control on the brain changes.

In their study, Mauras et al.along with assessing differences in brain structure among children with diabetes compared to healthy subjects, showed that these changes are related with cumulative hyperglycemia and notable changes in glucose levels; however, not correlated with hypoglycemia [8]. Marzelli et al. also showed that the volume of gray matter in areas such as bilateral lingual gyrus, fusiform gyrus, right parahippocampal gyrus, and cerebellum in are reversely associated with HbA1C levels.

Similarly, the volume of gray matter in areas of bilateral medial orbitofrontal, rectal gyri, and the anterior cingulate are inversely associated with the duration of diabetes. In addition, the volume of white matter in the left anterior frontal lobe has been shown to be negatively correlated with duration of disease [9]. Also, in the study of Barnea-Goraly et al. recurrent hyperglycemia in diabetic patients showed a significant correlation with structural changes in white matter. But, this correlation was not seen in cases of hypoglycemia.

Duration of diabetes revealed a significant inverse correlation with the size of axial diffusivity (AD) and radial diffusivity (RD) and a significant positive correlation with Fractional anisotropy (FA) values in widespread brain regions. No significant difference has been seen in the volume of white matter in children with diabetes who experience severe hypoglycemia and seizures and the rest of diabetics without such experiences. On the other hand, younger ages of developing diabetes showed a positive correlation with lower FA and higher RD [10]. In another study with a similar goal, a history of three or more severe attacks of hyperglycemia revealed a significant correlation with reduced anisotropy, increased diffusivity in the superior parietal lobule, and increased diffusivity in the hippocampus [19].

In the study of Aye et al., a direct and significant correlation between HbA1C and RD was found which was observable in the inferior fronto –occipital fasciculi, uncinated fasciculi, subgenual WM, anterior forceps, right internal capsule, superior middle, inferior temporal gyri, splenium of corpus callusome, superior longitudinal fasciculi, and occipital WM. In this study, a negative correlation between the level of HbA1C and FA in right internal capsule, anterior forceps, inferior fronto-occipital fasciculi and splenium of CC have also been reported [11].

In another study, Aye et al. showed that diabetic children with a history of seizures developed a reduced volume of gray and white matters compared to those with diabetes who had no history of seizures. But, the history of seizures did not show any impact on the volume of the hippocampus. As well, HbA1C levels showed no correlation with changes in the volume of brain structures [12]. Also, in a two-year cohort study conducted on diabetic patients Perantie et al. concluded that patients with recurrent hyperglycemia episodes and a more intense reduced volume of gray matter. Moreover, patients who experience severe recurrent hypoglycemia were at higher risk of reducing the volume of white matter in areas of the occipital and parietal [13].

In another study on diabetic children, he also reported that patients with history of hypoglycemia had a lower volume of gray matter in the superior temporal, occipital cortex, and left inferior occipital cortex compared to the group without a history of hypoglycemia.

In diabetes, recurrent hyperglycemia was found to be associated with less gray matter volume in the right cuneus and precuneus. As well, it was associated with higher volume of gray matter in the right frontal middle gyrus and lower volume of white matter in the right superior parietal (WM). Also, its indicated that the early onset of the disease was related with greater white matter volume in the left precuneus region [14]. In the study of Hershey et al., the number of severe hypoglycemic attacks was significantly correlated with the enlargement of hippocampal gray matter volume. This correlation was more highlighted in the subgroup with 3 or more severe attacks. This study didn’t find any pattern indicating the changes in the hippocampal gray matter volume due to chronic hyperglycemia [16].

Northamet al. also claimed in their research that hypoglycemia is a predictor of gray matter loss in the thalamus. This survey also revealed an association between poorly controlled metabolic and T2 reduction in the thalamus [17].

## Discussion and conclusion

Several evidence is available indicating the structural changes in the central nervous system as a complication of diabetes in adults [20]. Also, in some studies, vascular complications in the context of diabetes is considered as one of the factors contributing to brain changes in patients with diabetes which can increase the risk of some disorders such as stroke [21]. Incidentally, disorders such as dementia, as one of the neurological complications of diabetes, are linked to this problem [22]. But, the analysis of structural changes in the brains of diabetic children is a subject that has been discussed in recent years and various studies have been conducted in this regard. However, as may be expected fewer literature is available on the brain structure complications in pediatric diabetes compared to in adults.

The authors of the present study have attempted to make a comprehensive review of the efforts of other researchers to explore the association between diabetes and brain structural changes. Of course, neurological changes in the context of some severe complications of diabetes, such as ketoacidosis have been the subject of several studies which were not included in this study. This is mainly because the subjects in these studies were merely children with diabetes who had these complications and may not be a perfect example of the diabetic children population.

As can be seen in these studies, a huge proportion of structures of the central nervous system have been affected by diabetes that include different areas of gray and white matters. Of these studies, only two longitudinal studies have evaluated the developmental changes in the central nervous system in diabetic patients over times, which have not reported consistent results with together. Other studies reviewed in this paper are cross-sectional and case–control studies which the majority have reported various brain structural differences in children and adolescents with diabetes compare to healthy subjects.

This could explain the cognitive changes in diabetic children who have been investigated in several studies. Also, newer researches on children and adolescents with diabetes conducted by the application of functional brain imaging techniques have presented new evidence regarding this association.

Of other important aspects that should be taken into account in these studies is the association of glycemic control and the structural changes in the central nervous system of children and adolescents with diabetes. In the majority of these studies, it has become clear that high glycemic changes, especially recurrent hyperglycemic attacks are very seriously associated with structural changes in the brain.

Now, if one takes into consideration the fact that a significant proportion of children and adolescents with diabetes lack good glycemic control parameters [23], it must be accepted that many children and adolescents with diabetes should suffer from structural changes in the brain and, consequently, serious cognitive changes during the course of their disease.

This issue must be well taken into account and the families of children with diabetes must be well understood by the health care providers.

One of the considerations that must be made when reviewing these studies is that apart from two studies on children and adolescents with type 2 diabetes, other studies had been conducted on patients with type 1 diabetes. Moreover, none of the studies have compared the changes in brain structure in children and adolescents with type 1diabetes and those with type 2, and in this respect, the difference between these two types of diseases affecting the central nervous system is not well illustrated. On the other hand, given the growing trend of developing type 2 diabetes among adolescents, further evidence indicating the association between type 2 diabetes and the structural changes of brain is needed [24].

Of the limitations observable in related studies are the lack of prospective studies exploring this association. Surely, design and implementation of prospective studies can provide answers to serious questions in this area including whether adequate glycemic control after the structural changes of the nervous system can resolve the changes or not, and or the brain structural changes seen in children and adolescents with diabetes compared to healthy peers is merely due to diabetes or other effective factors have created such differences. The answers to these questions can positively aid therapists to develop medical guidelines to prevent or resolve the changes in nervous structure.

## Abbreviations

AD: axial diffusivity
CC: corpus callosum
CSF: Cerebro Spinal Fluid
FA: Functional anisotropy
MRI: Magnetic Resonance Imaging
RD: Radial diffusivity
WM: White Matter
